# Chloridonitros­yl[*meso*-5,10,15,20-tetra­kis­(*p*-tol­yl)porphyrinato-κ^4^ *N*,*N*′,*N*′′,*N*′′′]osmium(II) tetra­hydro­furan tetra­solvate

**DOI:** 10.1107/S1600536811001401

**Published:** 2011-01-22

**Authors:** Li Chen, Nan Xu, Douglas R. Powell, George B. Richter-Addo

**Affiliations:** a Department of Chemistry and Biochemistry, University of Oklahoma, 101 Stephenson Parkway, Norman, OK 73019-5251, USA

## Abstract

The title compound, [OsCl(NO)(C_48_H_36_N_4_)]·4C_4_H_8_O, is a six-coordinate osmium(II) porphyrin complex with nitrosyl (NO) and chloride (Cl) ligands *trans* to each other in an octa­hedral geometry. The metal complex lies on a fourfold rotation axis that passes through the Os, N, O and Cl atoms. The NO and Cl ligands are disordered in an 0.511 (12):0.486 (12) ratio.

## Related literature

For related osmium nitrosyl porphyrin derivatives, see: Cheng *et al.* (2001 ▶); Lee *et al.* (2001 ▶). For the synthesis, see: Cheng *et al.* (1998 ▶).
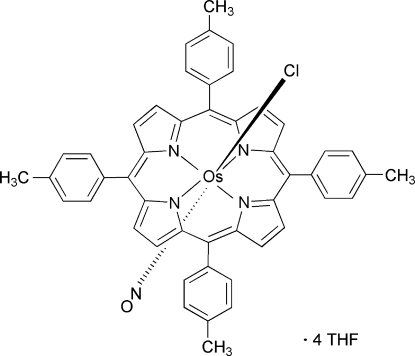


## Experimental

### 

#### Crystal data

[OsCl(NO)(C_48_H_36_N_4_)]·4C_4_H_8_O
*M* *_r_* = 1212.88
Tetragonal, 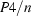

*a* = 16.905 (2) Å
*c* = 9.6220 (19) Å
*V* = 2749.8 (8) Å^3^
*Z* = 2
Mo *K*α radiation
μ = 2.42 mm^−1^
*T* = 188 K
0.62 × 0.58 × 0.52 mm

#### Data collection

Siemens P4 diffractometer
Absorption correction: ψ scan (North *et al.*, 1968 ▶) *T*_min_ = 0.315, *T*_max_ = 0.366
8309 measured reflections
2859 independent reflections
2749 reflections with *I* > 2σ(*I*)
*R*_int_ = 0.024
3 standard reflections every 97 reflections  intensity decay: 6.7%

#### Refinement

*R*[*F* ^2^ > 2σ(*F* ^2^)] = 0.022
*wR*(*F* ^2^) = 0.058
*S* = 0.97
2859 reflections
187 parameters
1 restraint
H-atom parameters constrained
Δρ_max_ = 0.59 e Å^−3^
Δρ_min_ = −1.46 e Å^−3^

### 

Data collection: *XSCANS* (Siemens, 1994 ▶); cell refinement: *XSCANS*; data reduction: *SHELXTL* (Sheldrick, 2008 ▶); program(s) used to solve structure: *SHELXTL*; program(s) used to refine structure: *SHELXTL*; molecular graphics: *SHELXTL*; software used to prepare material for publication: *SHELXTL*.

## Supplementary Material

Crystal structure: contains datablocks I, global. DOI: 10.1107/S1600536811001401/ng5100sup1.cif

Structure factors: contains datablocks I. DOI: 10.1107/S1600536811001401/ng5100Isup2.hkl

Additional supplementary materials:  crystallographic information; 3D view; checkCIF report

## Figures and Tables

**Fig. 1.**
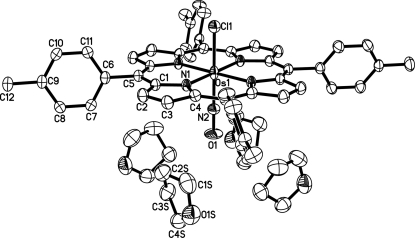
The molecular structure of (TTP)Os(NO)Cl.4THF. Displacement ellipsoids are drawn at the 50% probability level. H atoms are omitted for clarity.

## supplementary crystallographic information

### Comment

Six-coordinate osmium nitrosyl porphyrin complexes have been prepared as potential heme-NO structural models (Cheng *et al.* 2001 and Lee *et al*. 2001). Compared to their iron derivatives, osmium nitrosyl porphyrin compounds are more thermally stable and more easily characterized spectroscopically. In this paper, we report the structure of (chloro)(nitrosyl)(*meso*-5,10,15,20-tetrakis(*p*-tolyl)porphyrinato)osmium with four molecules of tetrahydrofuran as the solvate.

The metal complex is found to sit on a crystallographic 4 axis. The nitrosyl and chloride ligands are disordered across the porphyrin plane. The occupancies of the nitrosyl and chloride ligands refine to 0.511 (12) and 0.486 (12) for the unprimed and primed atoms, respectively. The molecular structure of (TTP)Os(NO)Cl is shown in Figure 1. The nitrosyl group binds to the osmium center through its nitrogen atom, exhibiting a linear Os—N—O conformation (180°). The average Os—N_p_ distance is 2.0622 (19) Å. The average Os—N(O) distance is 1.80 (3) Å and the average Os—Cl distance is 2.208 (8) Å. The average N—O distance is 1.244 (2) Å.

### Experimental

The titled compound was obtained as a sideproduct during the preparation of (TTP)Os(NO)(Me) from the reaction of [(TTP)Os(NO)]PF_6_ with MeMgCl in THF (Cheng *et al.* 1998), and crystals were obtained by slow evaporation of a THF-hexane (1:1) solution at room temperature.

### Refinement

The hydrogen atoms were placed in calculated positions with C—H = 0.95 Å for aromatic carbons, 0.99 Å for methylene carbons and 0.98 Å for methyl carbons and were refined using a riding model with *U*_iso_ = 1.2 *U*_eq_(C) for phenyl H atoms, *U*_iso_ = 1.5 *U*_eq_(C) for methyl H atoms. The compound was made with Cl on one side of the porphyrin ring and NO on the other side of the ring. In the crystal structure, both ligands appeared to be on both sides of the ring. The Cl on one side of the ring was matched with the NO on the opposite side of the ring in the model. Because the Cl and NO groups were on a 4-fold axis, their occupancies from the two sets of ligands were set to sum to 0.25.

### Crystal data

**Table:**

[OsCl(NO)(C_48_H_36_N_4_)]·4C_4_H_8_O	*D*_x_ = 1.465 Mg m^−^^3^
*M**_r_* = 1212.88	Mo *K*α radiation, λ = 0.71073 Å
Tetragonal, *P*4/*n*	Cell parameters from 41 reflections
Hall symbol: -P 4a	θ = 6.9–12.4°
*a* = 16.905 (2) Å	µ = 2.42 mm^−^^1^
*c* = 9.6220 (19) Å	*T* = 188 K
*V* = 2749.8 (8) Å^3^	Block, purple
*Z* = 2	0.62 × 0.58 × 0.52 mm
*F*(000) = 1240	

### Data collection

**Table:**

Siemens P4 diffractometer	2749 reflections with *I* > 2σ(*I*)
Radiation source: fine-focus sealed tube	*R*_int_ = 0.024
graphite	θ_max_ = 26.5°, θ_min_ = 2.1°
ω scans	*h* = −1→21
Absorption correction: ψ scan (North *et al.*, 1968)	*k* = −14→21
*T*_min_ = 0.315, *T*_max_ = 0.366	*l* = −12→12
8309 measured reflections	3 standard reflections every 97 reflections
2859 independent reflections	intensity decay: 6.7%

### Refinement

**Table:**

Refinement on *F*^2^	Secondary atom site location: difference Fourier map
Least-squares matrix: full	Hydrogen site location: inferred from neighbouring sites
*R*[*F*^2^ > 2σ(*F*^2^)] = 0.022	H-atom parameters constrained
*wR*(*F*^2^) = 0.058	*w* = 1/[σ^2^(*F*_o_^2^) + (0.040*P*)^2^ + 0.950*P*] where *P* = (*F*_o_^2^ + 2*F*_c_^2^)/3
*S* = 0.97	(Δ/σ)_max_ = 0.001
2859 reflections	Δρ_max_ = 0.59 e Å^−^^3^
187 parameters	Δρ_min_ = −1.46 e Å^−^^3^
1 restraint	Extinction correction: *SHELXTL* (Sheldrick, 2008), Fc^*^=kFc[1+0.001xFc^2^λ^3^/sin(2θ)]^-1/4^
Primary atom site location: structure-invariant direct methods	Extinction coefficient: 0.00138 (16)

### Special details

**Table:**

Refinement. Restraint: sump 0.25 0.0004 1 2 1 3 where the second and third item on the fvar instruction were occupancies of N2, O1, Cl1, and N2', O1', and Cl1', respectively.

### Fractional atomic coordinates and isotropic or equivalent isotropic displacement parameters (Å^2^)

**Table:**

	*x*	*y*	*z*	*U*_iso_*/*U*_eq_	Occ. (<1)
Os1	0.2500	0.2500	0.764201 (16)	0.02581 (8)	
N1	0.35454 (11)	0.31287 (11)	0.76484 (15)	0.0223 (3)	
C1	0.42941 (13)	0.28109 (14)	0.76543 (17)	0.0245 (4)	
C2	0.48571 (14)	0.34489 (15)	0.76709 (19)	0.0298 (5)	
H2	0.5417	0.3397	0.7685	0.036*	
C3	0.44474 (14)	0.41381 (15)	0.76625 (19)	0.0294 (5)	
H3	0.4666	0.4656	0.7661	0.035*	
C4	0.36169 (14)	0.39376 (13)	0.76565 (18)	0.0245 (4)	
C5	0.44808 (13)	0.20028 (14)	0.76528 (17)	0.0246 (4)	
C6	0.53430 (14)	0.17924 (14)	0.76365 (18)	0.0254 (4)	
C7	0.57898 (12)	0.19002 (13)	0.6443 (2)	0.0308 (4)	
H7	0.5545	0.2103	0.5629	0.037*	
C8	0.65901 (12)	0.17163 (13)	0.6424 (2)	0.0318 (5)	
H8	0.6883	0.1787	0.5591	0.038*	
C9	0.69700 (14)	0.14299 (15)	0.76017 (18)	0.0279 (5)	
C10	0.65179 (13)	0.13145 (14)	0.8786 (2)	0.0361 (5)	
H10	0.6761	0.1112	0.9602	0.043*	
C11	0.57146 (12)	0.14902 (14)	0.8803 (2)	0.0350 (5)	
H11	0.5417	0.1402	0.9625	0.042*	
C12	0.78439 (16)	0.12540 (19)	0.7586 (2)	0.0390 (6)	
H12A	0.7967	0.0865	0.8309	0.058*	
H12B	0.8140	0.1742	0.7763	0.058*	
H12C	0.7993	0.1041	0.6676	0.058*	
N2	0.2500	0.2500	0.5792 (16)	0.031 (4)	0.511 (12)
O1	0.2500	0.2500	0.4507 (15)	0.057 (2)	0.511 (12)
Cl1	0.2500	0.2500	0.9929 (8)	0.031 (2)	0.511 (12)
N2'	0.2500	0.2500	0.953 (3)	0.040 (8)	:0.486 (12)
O1'	0.2500	0.2500	1.0823 (14)	0.048 (2)	0.486 (12)
Cl1'	0.2500	0.2500	0.5339 (5)	0.0303 (18)	0.486 (12)
O1S	0.39867 (19)	0.45886 (16)	0.2341 (2)	0.0674 (7)	
C1S	0.3997 (2)	0.4362 (2)	0.3748 (3)	0.0680 (9)	
H1SA	0.3451	0.4329	0.4117	0.082*	
H1SB	0.4297	0.4750	0.4308	0.082*	
C2S	0.43853 (19)	0.3572 (2)	0.3803 (3)	0.0657 (8)	
H2SA	0.4198	0.3261	0.4610	0.079*	
H2SB	0.4968	0.3622	0.3847	0.079*	
C3S	0.4123 (3)	0.3206 (2)	0.2450 (3)	0.0567 (8)	
H3SA	0.4515	0.2817	0.2109	0.068*	
H3SB	0.3603	0.2944	0.2548	0.068*	
C4S	0.40732 (18)	0.39121 (18)	0.1500 (3)	0.0550 (7)	
H4SA	0.4559	0.3954	0.0931	0.066*	
H4SB	0.3614	0.3859	0.0867	0.066*	

### Atomic displacement parameters (Å^2^)

**Table:**

	*U*^11^	*U*^22^	*U*^33^	*U*^12^	*U*^13^	*U*^23^
Os1	0.01638 (8)	0.01638 (8)	0.04466 (12)	0.000	0.000	0.000
N1	0.0171 (8)	0.0180 (8)	0.0317 (8)	−0.0005 (7)	−0.0002 (5)	0.0002 (5)
C1	0.0190 (10)	0.0258 (11)	0.0287 (9)	−0.0003 (8)	−0.0006 (6)	−0.0003 (7)
C2	0.0227 (11)	0.0288 (12)	0.0381 (11)	−0.0019 (9)	−0.0003 (7)	−0.0016 (7)
C3	0.0249 (11)	0.0264 (11)	0.0369 (11)	−0.0050 (9)	0.0009 (7)	−0.0010 (7)
C4	0.0244 (11)	0.0202 (10)	0.0288 (9)	−0.0035 (8)	0.0008 (7)	−0.0008 (6)
C5	0.0195 (10)	0.0265 (11)	0.0278 (9)	0.0015 (8)	−0.0005 (6)	−0.0004 (7)
C6	0.0204 (10)	0.0231 (11)	0.0326 (10)	0.0001 (8)	−0.0017 (7)	−0.0027 (7)
C7	0.0261 (10)	0.0356 (11)	0.0308 (9)	0.0044 (9)	−0.0012 (7)	0.0059 (8)
C8	0.0259 (10)	0.0370 (12)	0.0324 (10)	0.0024 (9)	0.0043 (7)	0.0051 (8)
C9	0.0207 (11)	0.0267 (12)	0.0363 (11)	0.0023 (9)	−0.0014 (7)	−0.0016 (7)
C10	0.0283 (11)	0.0491 (14)	0.0309 (10)	0.0070 (10)	−0.0048 (8)	0.0054 (9)
C11	0.0259 (10)	0.0500 (14)	0.0292 (10)	0.0044 (9)	0.0020 (8)	0.0035 (9)
C12	0.0240 (12)	0.0486 (17)	0.0443 (13)	0.0071 (11)	−0.0008 (8)	0.0045 (9)
N2	0.034 (4)	0.034 (4)	0.025 (11)	0.000	0.000	0.000
O1	0.077 (5)	0.077 (5)	0.016 (5)	0.000	0.000	0.000
Cl1	0.0298 (11)	0.0298 (11)	0.032 (6)	0.000	0.000	0.000
N2'	0.039 (5)	0.039 (5)	0.04 (2)	0.000	0.000	0.000
O1'	0.065 (4)	0.065 (4)	0.015 (5)	0.000	0.000	0.000
Cl1'	0.0354 (10)	0.0354 (10)	0.020 (5)	0.000	0.000	0.000
O1S	0.0723 (18)	0.0438 (14)	0.0861 (17)	0.0008 (13)	−0.0012 (11)	0.0023 (9)
C1S	0.0578 (19)	0.077 (2)	0.0693 (19)	0.0008 (16)	0.0034 (15)	−0.0246 (17)
C2S	0.0583 (19)	0.089 (2)	0.0504 (16)	0.0095 (16)	−0.0073 (13)	0.0021 (15)
C3S	0.060 (2)	0.0456 (19)	0.0643 (19)	0.0071 (17)	−0.0055 (12)	−0.0029 (11)
C4S	0.0531 (17)	0.0637 (19)	0.0480 (14)	−0.0037 (14)	−0.0013 (12)	0.0037 (12)

### Geometric parameters (Å, °)

**Table:**

Os1—N2	1.780 (16)	C9—C12	1.507 (3)
Os1—N2'	1.81 (3)	C10—C11	1.390 (3)
Os1—N1^i^	2.0622 (19)	C10—H10	0.9500
Os1—N1	2.0622 (19)	C11—H11	0.9500
Os1—Cl1	2.201 (8)	C12—H12A	0.9800
Os1—Cl1'	2.216 (8)	C12—H12B	0.9800
N1—C4	1.373 (3)	C12—H12C	0.9800
N1—C1	1.375 (3)	N2—O1	1.237 (16)
C1—C5	1.402 (3)	N2'—O1'	1.25 (2)
C1—C2	1.439 (3)	O1S—C1S	1.407 (4)
C2—C3	1.355 (4)	O1S—C4S	1.408 (4)
C2—H2	0.9500	C1S—C2S	1.488 (5)
C3—C4	1.444 (3)	C1S—H1SA	0.9900
C3—H3	0.9500	C1S—H1SB	0.9900
C4—C5^i^	1.393 (3)	C2S—C3S	1.508 (4)
C5—C6	1.500 (3)	C2S—H2SA	0.9900
C6—C11	1.384 (3)	C2S—H2SB	0.9900
C6—C7	1.386 (3)	C3S—C4S	1.506 (4)
C7—C8	1.388 (3)	C3S—H3SA	0.9900
C7—H7	0.9500	C3S—H3SB	0.9900
C8—C9	1.389 (3)	C4S—H4SA	0.9900
C8—H8	0.9500	C4S—H4SB	0.9900
C9—C10	1.386 (3)		
			
N2—Os1—N1	90.17 (4)	C11—C10—H10	119.4
N2'—Os1—N1	89.83 (4)	C6—C11—C10	120.9 (2)
N1—Os1—N1^ii^	90.0	C6—C11—H11	119.6
N2'—Os1—Cl1'	180.000 (5)	C10—C11—H11	119.6
N1—Os1—Cl1'	90.17 (4)	C9—C12—H12A	109.5
C4—N1—C1	107.95 (19)	C9—C12—H12B	109.5
C4—N1—Os1	126.07 (15)	H12A—C12—H12B	109.5
C1—N1—Os1	125.98 (15)	C9—C12—H12C	109.5
N1—C1—C5	126.0 (2)	H12A—C12—H12C	109.5
N1—C1—C2	108.4 (2)	H12B—C12—H12C	109.5
C5—C1—C2	125.6 (2)	O1—N2—Os1	180.000 (2)
C3—C2—C1	107.8 (2)	O1'—N2'—Os1	180.000 (4)
C3—C2—H2	126.1	C1S—O1S—C4S	109.3 (2)
C1—C2—H2	126.1	O1S—C1S—C2S	106.5 (2)
C2—C3—C4	107.2 (2)	O1S—C1S—H1SA	110.4
C2—C3—H3	126.4	C2S—C1S—H1SA	110.4
C4—C3—H3	126.4	O1S—C1S—H1SB	110.4
N1—C4—C5^i^	126.2 (2)	C2S—C1S—H1SB	110.4
N1—C4—C3	108.6 (2)	H1SA—C1S—H1SB	108.6
C5^i^—C4—C3	125.2 (2)	C1S—C2S—C3S	102.0 (3)
C4^ii^—C5—C1	125.8 (2)	C1S—C2S—H2SA	111.4
C4^ii^—C5—C6	117.5 (2)	C3S—C2S—H2SA	111.4
C1—C5—C6	116.7 (2)	C1S—C2S—H2SB	111.4
C11—C6—C7	118.2 (2)	C3S—C2S—H2SB	111.4
C11—C6—C5	121.33 (18)	H2SA—C2S—H2SB	109.2
C7—C6—C5	120.45 (18)	C4S—C3S—C2S	102.4 (3)
C6—C7—C8	120.84 (19)	C4S—C3S—H3SA	111.3
C6—C7—H7	119.6	C2S—C3S—H3SA	111.3
C8—C7—H7	119.6	C4S—C3S—H3SB	111.3
C7—C8—C9	121.17 (19)	C2S—C3S—H3SB	111.3
C7—C8—H8	119.4	H3SA—C3S—H3SB	109.2
C9—C8—H8	119.4	O1S—C4S—C3S	107.5 (2)
C10—C9—C8	117.7 (2)	O1S—C4S—H4SA	110.2
C10—C9—C12	121.40 (18)	C3S—C4S—H4SA	110.2
C8—C9—C12	120.91 (18)	O1S—C4S—H4SB	110.2
C9—C10—C11	121.2 (2)	C3S—C4S—H4SB	110.2
C9—C10—H10	119.4	H4SA—C4S—H4SB	108.5
			
N2—Os1—N1—C4	90.28 (13)	C2—C3—C4—C5^i^	179.72 (17)
N2'—Os1—N1—C4	−89.72 (13)	N1—C1—C5—C4^ii^	−0.5 (3)
N1^i^—Os1—N1—C4	0.11 (17)	C2—C1—C5—C4^ii^	179.04 (17)
N1^ii^—Os1—N1—C4	−179.55 (11)	N1—C1—C5—C6	179.03 (15)
Cl1—Os1—N1—C4	−89.72 (13)	C2—C1—C5—C6	−1.4 (3)
Cl1'—Os1—N1—C4	90.28 (13)	C4^ii^—C5—C6—C11	−73.1 (3)
N2—Os1—N1—C1	−90.17 (13)	C1—C5—C6—C11	107.3 (2)
N2'—Os1—N1—C1	89.83 (13)	C4^ii^—C5—C6—C7	107.2 (2)
N1^i^—Os1—N1—C1	179.66 (10)	C1—C5—C6—C7	−72.4 (3)
N1^ii^—Os1—N1—C1	0.00 (16)	C11—C6—C7—C8	−0.6 (3)
Cl1—Os1—N1—C1	89.83 (13)	C5—C6—C7—C8	179.2 (2)
Cl1'—Os1—N1—C1	−90.17 (13)	C6—C7—C8—C9	−0.9 (3)
C4—N1—C1—C5	179.82 (16)	C7—C8—C9—C10	1.7 (3)
Os1—N1—C1—C5	0.2 (2)	C7—C8—C9—C12	−178.2 (2)
C4—N1—C1—C2	0.18 (19)	C8—C9—C10—C11	−0.9 (3)
Os1—N1—C1—C2	−179.44 (12)	C12—C9—C10—C11	178.9 (2)
N1—C1—C2—C3	−0.5 (2)	C7—C6—C11—C10	1.3 (3)
C5—C1—C2—C3	179.82 (17)	C5—C6—C11—C10	−178.5 (2)
C1—C2—C3—C4	0.6 (2)	C9—C10—C11—C6	−0.5 (4)
C1—N1—C4—C5^i^	179.94 (16)	C4S—O1S—C1S—C2S	20.6 (4)
Os1—N1—C4—C5^i^	−0.4 (2)	O1S—C1S—C2S—C3S	−33.6 (4)
C1—N1—C4—C3	0.22 (19)	C1S—C2S—C3S—C4S	32.9 (4)
Os1—N1—C4—C3	179.84 (11)	C1S—O1S—C4S—C3S	1.3 (4)
C2—C3—C4—N1	−0.6 (2)	C2S—C3S—C4S—O1S	−22.0 (4)

Symmetry codes: (i) −*y*+1/2, *x*, *z*; (ii) *y*, −*x*+1/2, *z*.
